# Complete mitochondrial genome of the marine mysid *Siriella* sp. (Crustacea, Mysida, Mysidae)

**DOI:** 10.1080/23802359.2019.1636725

**Published:** 2019-07-12

**Authors:** Do-Hee Lee, Bo-Mi Kim, Jae-Sung Rhee

**Affiliations:** a Department of Marine Science, College of Natural Sciences, Incheon National University, Incheon, South Korea;; b Unit of Polar Genomics, Korea Polar Research Institute, Incheon, South Korea;; c Research Institute of Basic Sciences, Incheon National University, Incheon, South Korea

**Keywords:** Mysida, marine mysid, *Siriella*, mitogenome

## Abstract

The complete mitochondrial genome of the marine mysid, *Siriella* sp. was obtained by conventional polymerase chain reaction (PCR) method. Total length of *Siriella* sp. mitochondrial genome was 14,706 bp, with the base composition of 27% A, 21% C, 22% G, and 30% T with a high AT bias of 57%. The mitogenome of *Siriella* sp. contained 13 protein-coding genes (PCGs), 22 transfer RNA (tRNA) genes, 2 ribosomal RNA (rRNA) genes, and a putative control region. Maximum likelihood method-based phylogenetic reconstruction suggested the evolutionary relationship to other mysids within the order Mysida. Since Mysida contains numerous species across a wide range of water habitats, this information will provide an essential molecular reference to elucidate biogeography, phylogenetic distance, and evolutionary diversity in mysids. This is the first mitogenome information in the genus *Siriella*.

Mysids are small, shrimp-like omnivorous crustaceans and are ecologically important as a valuable food source for a variety of coastal organisms. Entire larval development in a marsupium and release of free-swimming juveniles are fascinating characteristics of mysids (Wittmann 1984). Species of the order Mysida are distributed worldwide in intertidal zones from tropical to temperate coastal waters with high species diversity. Mysids have been recognized as a model species for toxicity test, ecotoxicology, and environmental research due to several promising features, which include small size, fast growth rate with high productivity, sensitivity, high adaptability to diverse ranges of seawater conditions, feasibility of multi-generation test, good representatives of shrimp, and ease of aquaculture in the laboratory (Verslycke et al. 2004; Hirano et al. 2009; Do et al. 2018; Haque et al. 2018; Kim et al. 2018; Min et al. 2018). Despite the high species diversity and abundance of the order Mysida, only several studies have been investigated on characteristics and organization of whole mitogenomes (Shen et al. 2015; Song et al. 2016). Previously, 77 species have been recorded in the genus *Siriella* Dana, 1850 in the world (Murano and Fukuoka 2008), and the genus is divided into 9 groups with 5 subgroups that comprise 8 known species in the coastal regions of Korea (Lee et al. 2017). *Siriella* sp. was also suggested as a suitable model species for ecotoxicology (Pérez and Beiras 2010). However, mitogenome analysis and studies on in-depth molecular phylogenetic relationship have yet to be conducted. Since high-level taxonomy of the order Mysida is complex due to morphologically heterogeneous characteristics, accumulation of the mitogenome information is strongly needed to explain molecular phylogeny, evolution, and genetic diversity of mysids.

In this study, we analyzed the mitogenome of the marine mysid, *Siriella* sp. as the first report in the genus *Siriella* (Accession no. MK948869). Specimen of *Siriella* sp. was sampled from the coastal region of Dolsando, Yeosu, South Korea (34°35′32.9″N 127°45′35.5″E) and was preserved in 100% ethanol. The voucher specimen was stored in the Research Institute of Basic Sciences of Incheon National University (Specimen ID: 201806-Mysid038). Genomic DNA was isolated from the whole body of the single specimen by using the DNeasy Blood and Tissue kit (Qiagen, Hilden, Germany). The genomic DNA was qualified and quantified by using a Qubit 4 Fluorometer (Thermo Fisher Scientific, Waltham, MA, USA). The species identity was checked by morphological characteristics such as uropod, male pleopods, and telson (Murano and Fukuoka 2008) and sequence analysis of mitochondrial DNA cytochrome oxidase 1 (*co1*) and cytochrome b (*cytb*) using universal primer sets (Palumbi and Benzie 1991; Folmer et al. 1994). Whole mitogenome of *Siriella* sp. was sequenced by Sanger-sequencing with specific primers based on the *co1* and *cytb* sequences. Essential PCGs were annotated by using the MITOS web-based software (Bernt et al. 2013) and detailed annotation was performed with NCBI-BLAST (http://blast.ncbi.nlm.nih.gov).

The complete mitogenome of *Siriella* sp. was 14,706 bp in length and contained the typical set of 13 PCGs, 22 tRNAs, 2 rRNAs, and a putative AT-rich region. Overall, gene order and content of *Siriella* sp. mitogenome was similar to those of mysids, *Neomysis orientalis* and *N. japonica*, with inversions of several PCGs (Shen et al. 2015; Song et al. 2016), but the structure was quite different to other crustaceans as shown in *N. orientalis* (Shen et al. 2015). The nucleotide composition of *Siriella* sp. mitogenome is slightly biased toward A + T nucleotides, accounting for 27% A, 21% C, 22% G, and 30% T. Phylogenetic relationship was analyzed with the nucleotide sequences of cytochrome c oxidase subunit I (*CO1*) gene of mysids registered at GenBank (Figure 1), as only limited and partial genomic information of mysid mitogenomes is available as yet. By partitioned maximum likelihood analysis, the *Siriella* sp. mitogenome was clustered into a clade with *S. thompsonii* with high support value. Previously, phylogenetic analysis based on nuclear 18S ribosomal RNA sequences suggested that the subfamily Siriellinae is monophyletic (Remerie et al. 2004). Although the overall phylogenetic distance was relatively consistent with the traditional taxonomy and the Euphausiacea mitogenomes (North Pacific krill *Euphausia pacifica* and Antarctic krill *Euphausia superba*) were clearly recovered as an outgroup, we assume that more sequence information of the whole mitogenome should be accumulated to clarify the correlation between morphometric parameters and molecular distances in mysids. In conclusion, the *Siriella* sp. mitogenome will provide essential information to elucidate geographical distribution, phylogenetic relationship, and molecular evolution of the genus *Siriella* and the order Mysida.

**Figure 1.**
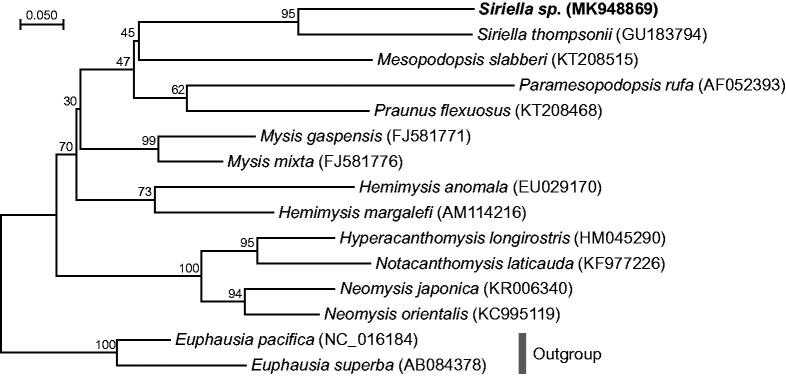
Maximum-likelihood (ML) phylogeny of the order Mysida based on the nucleotide sequences of cytochrome c oxidase subunit I (*CO1*) gene. Euphausiacea mitogenomes (North Pacific krill *Euphausia pacifica* and Antarctic krill *Euphausia superba*) were used as an outgroup for tree rooting. ML analysis was performed in RA + ML 8.2.10 using the mtREVþG model (Stamatakis 2014). Numbers on the branches indicate ML bootstrap percentages (1000 replicates). DDBJ/EMBL/Genbank accession numbers for published sequences are incorporated.
